# Black plastic mulch combined with summer cover crop increases the yield and water use efficiency of apple tree on the rainfed Loess Plateau

**DOI:** 10.1371/journal.pone.0185705

**Published:** 2017-09-28

**Authors:** Wei Zheng, Meijuan Wen, Zhiyuan Zhao, Jie Liu, Zhaohui Wang, Bingnian Zhai, Ziyan Li

**Affiliations:** 1 College of Resources and Environment, Northwest A&F University, Yangling, China; 2 Key Laboratory of Plant Nutrition and the Agri-environment in Northwest China, Ministry of Agriculture, Yangling, China; Instituto Agricultura Sostenible, SPAIN

## Abstract

Water deficit significantly limits dryland rainfed fruit production, so increasing water conservation is crucial for improving fruit productivity in arid and semiarid areas. In this study, we tested two treatments in an apple orchard: 1) PC treatment comprising black plastic mulch (BPM) (in-row) with weed control (inter-row); 2) and PGC treatment comprising BPM (in-row) combined with a summer cover crop (inter-row) of rape (*Brassica campestris* L.), which was sown in mid-June and was living from July to September. Under PGC, the inter-row soil water storage increased by 17.9% and 11.5% compared with PC after the harvest in 2013 and 2014, respectively, but there was no significant increase in 2015. The evapotranspiration (ET) from the inter-row areas during the cover crop period was lower under PGC than PC in 2013 (19.6%), 2014 (11.3%), and 2015 (13.3%). However, the differences in the total ET from the inter-row areas between the two treatments were not obvious, and the total ET from in-row areas was higher under PGC than PC due to the increased water uptake by apple trees under PGC. The apple yield, water use efficiency during the cover crop period (WUEg) and total water use efficiency (WUE) fluctuated during the experimental years. Compared with PC, the apple yield increased by 14.1%, 18.8%, and 26.7% under PGC in 2013, 2014, and 2015, respectively. In addition, the WUEg was 26.4%, 24.7%, and 32.7% higher under PGC compared with PC in 2013, 2014, and 2015, respectively. Thus, the WUE under PGC was 13.8% and 11.7% higher than that under PC in 2013 and 2014, respectively, but the difference was not significant in 2015 (*p* = 0.0527). Thus, BPM combined with a summer cover crop is recommended for decreasing the summer ET and promoting apple production in rainfed dryland areas where the rainy season is usually the hot season.

## Introduction

Black plastic mulch (BPM) is a popular agricultural treatment in dryland areas that experience water shortages and rainfall fluctuations, and it is commonly employed by farmers and orchardists for weed control and water conservation due to its opaqueness and imperviousness [1,2,3]. In addition, compared with other mulch materials (e.g., wheat straw, wood, and sawdust), BPM is more effective at decreasing evaporation as well as being economical, easy to obtain, and long-lasting. Thus, it has become increasingly popular for fruit production in arid and semiarid regions throughout the world.

The Loess Plateau is the largest dryland rainfed agricultural area in China, and it is also the biggest apple production area in China with 28.09% and 25.73% of the apple cultivation area and total yield, respectively [4]. Water deficit is a major limitation on apple production in this region. The Loess Plateau is dominated by the monsoon climate where 60% of the annual precipitation always occurs during the hot summer (July to September), but much of this is lost via evaporation due to the high temperatures during this period. Thus, BPM is applied extensively in many orchards on the Loess Plateau to decrease evaporation and conserve water to support apple tree growth. However, the water use efficiency (WUE) of trees is still low despite the good performance of BPM because of the poor soil fertility. The soil organic matter contents of most apple orchards on the Loess Plateau is usually in the range of 1.0–1.5%, which is much lower than that in American apple orchards (> 2.0%) [5, 6].

Cover crops are used widely in fruit orchards to increase the soil fertility. The many benefits of cover crops include weed control, decreased soil erosion, enhancement of the soil organic matter content, improved soil enzyme activities, increased water infiltration, and improvements to the soil structure and micro-ecological environment [7,8,9,10,11,12]. Furthermore, cover crops can increase the soil water content [13] and decrease the losses of soil water in some situations [14,15]. Thus, we hypothesized that the combination of BPM with a cover crop might be more effective at decreasing evaporation than the application of BPM (in-row) alone with bare soil (inter-row) if the cover crop performed well and apple tree growth was not influenced by competition for water with the cover crop. Many studies have investigated the separate effects of BPM or a cover crop on the water regimes in fruit orchards [1,15,16,17,18], but few data are available regarding the combined effect of BPM and a cover crop on water conservation in apple orchards on the Loess Plateau. In addition, it is unclear whether the competition for water between cover crops and apple trees might be mitigated by adjusting the sowing time of the cover crop.

To test these hypotheses and to understand the mechanism that explains the combined effect of BPM and a summer cover crop on soil water use in apple orchards, we tested two treatments in an apple orchard, where we determined the soil water storage (SWS), evapotranspiration (ET), apple yield, WUE, and soil temperature. We compared our results with the hypotheses to provide a theoretical basis for water conservation and apple production in local orchards as well as in orchards in similar dryland regions.

## Materials and methods

### Apple orchard description

**Fig 1:**
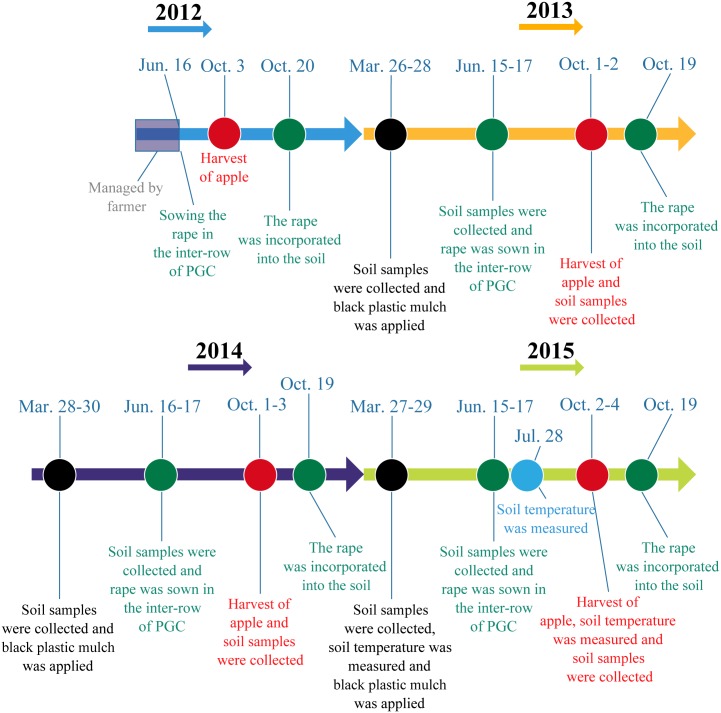
Chronological representation of soil collection and orchard management over the experimental period.

The orchard was established in 2003 at a typical site on the Loess Plateau located in Tianjiawa Village (109°52´E, 35°23´N; altitude 898.3 m), Baishui County, Shaanxi Province, China. The orchard was managed by farmers from 2003 to 2011, and BPM (in-row) with weed control (inter-row) was applied in the orchard. In mid-June 2012, the orchard was split into six plots, where three plots were selected randomly and rape (*Brassica campestris* L.) was sown in the inter-row areas between the apple trees (three plots selected). The study was conducted from 2013 to 2015 (Fig 1). The soil was silt loam (8% sand, 67% silt and 25% clay) and classified as Haplustalfs according to the USDA system of soil taxonomy. The topsoil had the following characteristics: pH = 8.11, available N = 10.28 mg kg^−1^, Olsen-phosphorus (P) = 6.91 mg kg^−1^, available potassium (K) = 101.40 mg kg^−1^, total N = 0.74 g kg^−1^, and organic matter content = 11.22 g kg^−1^. The summer is hot and moist in the study region, whereas the winter and early spring are always cold and dry. The annual average radiation was 5,360 MJ m^−2^ and the average number of annual sunlight hours was approximately 2,477 h. There were typically 207 frost-free days each year. Agriculture is completely dependent on natural precipitation in this region.

We state that no specific permissions were required for these locations/activities. We conform that the field studies did not involve endangered or protected species.

### Precipitation and air temperature

**Fig 2:**
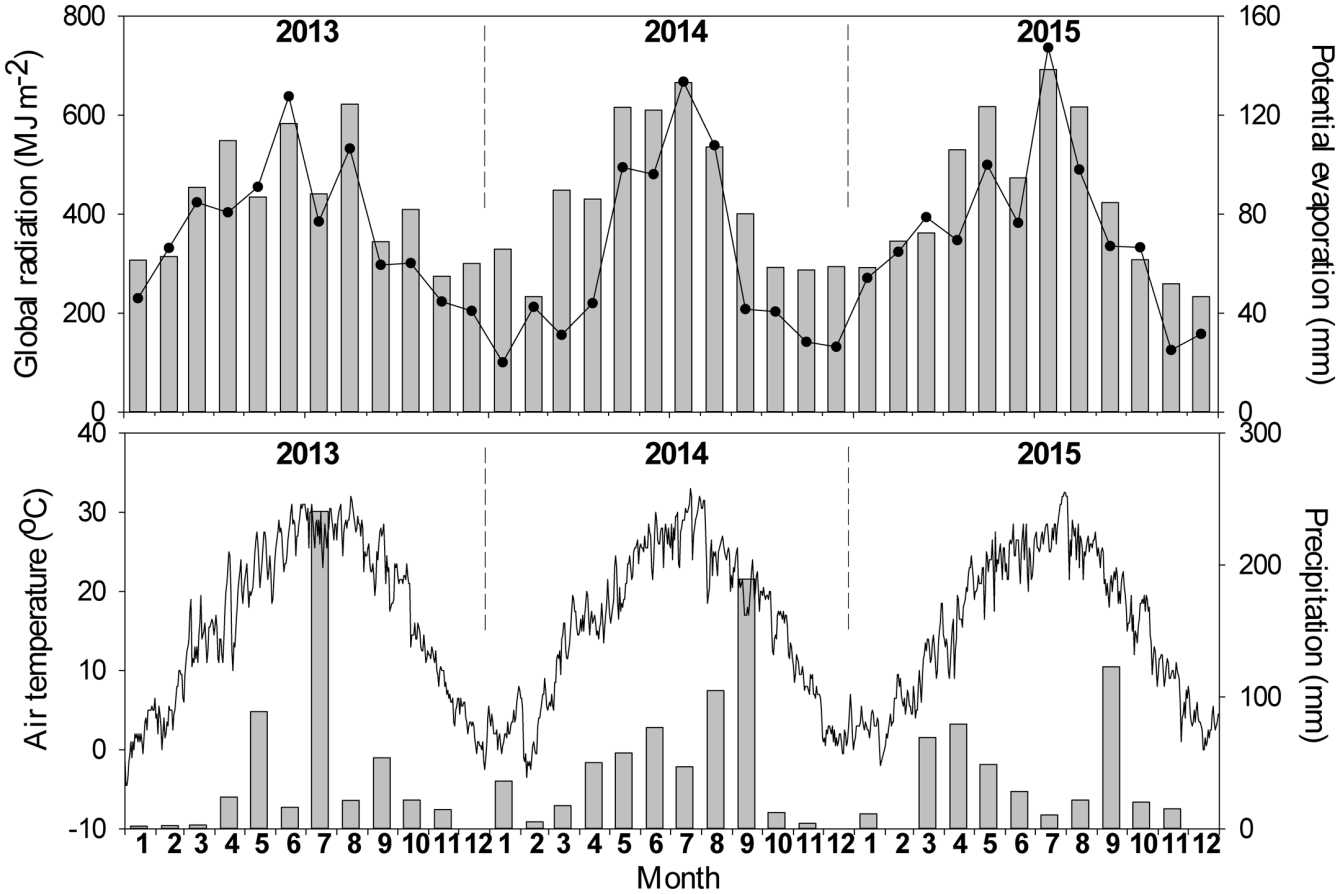
Monthly global radiation, potential evaporation, precipitation and mean daily air temperature of 2013, 2014 and 2015. Bars denote the monthly global radiation and precipitation, respectively. Lines represent the monthly potential evaporation and mean daily air temperature, respectively.

The monthly accumulated global radiation, potential evaporation, precipitation, and mean daily air temperature between 2013 and 2015 were recorded by a weather station (approximately 3,500 m from the apple orchard), and the values are shown in Fig 2.

The annual accumulated global radiation values in 2013, 2014, and 2015 were 5035, 5145, and 5155 MJ m^–2^, respectively (Fig 2). During the cover crop period (July–September), the accumulated global radiation values were 1408, 1603, and 1732 MJ m^–2^ in 2013, 2014, and 2015, respectively.

The annual potential evaporation levels in 2013, 2014, and 2015 were 885.2, 711.1, and 879.4 mm, respectively, and the potential evaporation levels during the cover crop period (July–September) were 243.0, 282.9, and 312.4 mm, (Fig 2).

The annual precipitation amounts in 2013, 2014, and 2015 were 489.6, 601.5, and 427.9 mm, respectively (Fig 1). The precipitation amounts during the cover crop period (July–September) were 316.2, 341.2, and 155.2 mm, which comprised 64.6%, 56.7%, and 36.3% of the annual precipitation in 2013, 2014, and 2015, respectively.

The daily mean temperature fluctuated greatly each year (Fig 2). The maximum mean daily air temperatures in 2013, 2014, and 2015 occurred on August 15 (32°C), July 21 (33°C), and July 30 (32.5°C), respectively. The minimum mean daily air temperatures in 2013, 2014, and 2015 occurred on January 2 (–4.5°C), February 7 (–3.5°C), and January 27 (–2.0°C), respectively. During the cover crop period (July–September), the average daily air temperatures were 26.5°C, 24.8°C, and 25.3°C in 2013, 2014, and 2015, respectively.

### Experimental design and treatments

**Fig 3:**
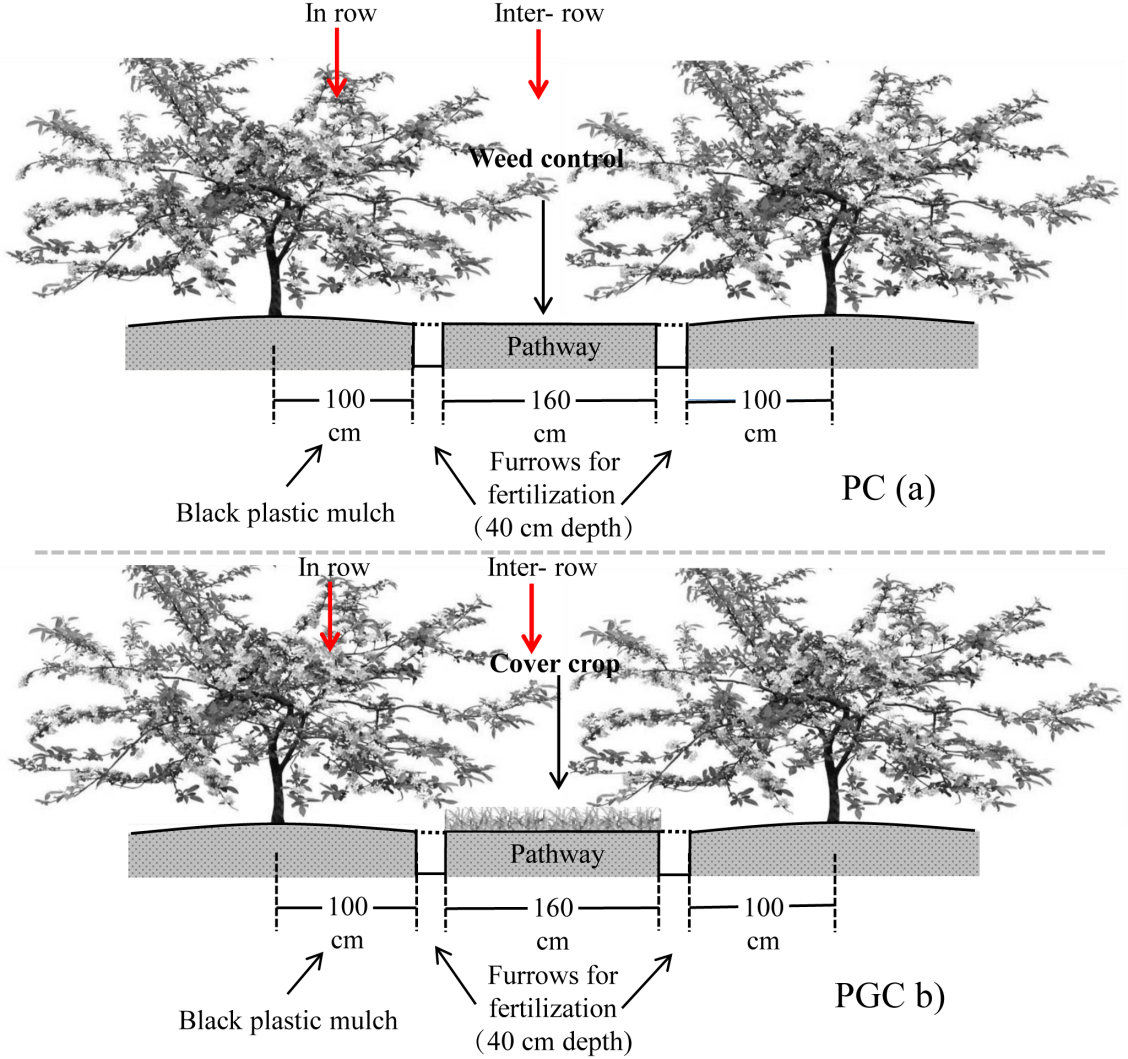
Schematic view of PC (black plastic mulch with weed control) and PGC (black plastic mulch combined with cover crop) treatments.

The plots were arranged in a randomized complete block design. Fuji apple trees (*Malus pumila* Mil.) were planted on M.26 rootstock with a spacing of 3 m within the rows and 4 m between the rows. Most of the apple orchards in this region employed BPM, so control treatments without cover crop and BPM, and individual applications of cover crop were not considered. Thus, two treatments with three replicates (20 apple trees each) were tested as follows: (1) PC treatment comprising BPM (in-row) with bare soil (inter-row) (Fig 3a), where polyethylene black plastic film with a thickness of 0.008 mm was applied manually (2 m wide, with 1 m either side of the trees) each year (Fig 1), with weed control each month by hoeing the inter-row areas (width = 1.6 m) where the residue was removed to leave bare soil, which were the same as the management practices employed by local farmers from 2003 to 2011. (2) PGC treatment comprising BPM combined with a summer cover crop (Fig 3b), where polyethylene black plastic film was used in the same manner as the PC treatment. To minimize the effects of water consumption by the cover crop on tree growth, the cover crop (rape, *Brassica campestris* L.) was sown (depth of about 2 cm) and harrowed in mid-June, and grown from July to September (rainy season), where the crop residues were incorporated into the soil to a depth of 20 cm using a rotary tiller in late October. The cover crop sowing rate was 7.5 kg per hectare of orchard. In previous years (2003–2011), chemical fertilizer and manure were applied every year, where the two treatments were applied at the same ratio. In late October during 2011, 300 kg N (urea, 46% N), 180 kg P_2_O_5_ (diammonium phosphate, 46% P_2_O_5_ and 18% N), 270 kg K_2_O (potassium sulfate, 50% K_2_O), and 22.5 t ha^–1^ pig manure were applied as basal fertilizer. The chemical fertilizer and manure were both applied in the furrows (Fig 3). The experiment commenced in mid-June 2012. The chemical fertilizer (210 kg N ha^–1^, 120 P_2_O_5_ ha^–1^, and 150 K_2_O ha^–1^) and manure (45 t ha^–1^) were applied as basal fertilizers in the orchard during late October in 2012, 2013 and 2014.

### Measurements and statistical analyses

SWS was measured at the bud formation stage (late March), before sowing the rape seed (mid-June), and after the harvest (early October) each year (Fig 1). Soil samples from each replicate were collected from 0–200 cm at 20 cm intervals using an open-faced bucket probe (diameter = 5 cm). Three soil cores were collected randomly under the black plastic film as well as from the inter-row areas in each replicate. The soil samples were taken to the laboratory to determine the gravimetric water content by oven drying at 105°C. SWS was determined as the sum of the SWS in each layer, which was calculated using the following formula [19]:

$$\text{Soil water storage of each layer}=\text{H }(\text{mm})\times \alpha \;(\text{g}\cdot {\text{cm}}^{-3})\times \beta \;(\%),$$

where H is the thickness of each layer (20 cm in our experiment), α is the bulk density of each layer, and β represents the gravimetric water content of each layer. The soil bulk density was measured as described by Liu et al. (2013), where it was 1.20, 1.39, 1.43, 1.31, 1.30, 1.27, 1.26, 1.25, 1.28, and 1.29 g cm^–3^ in each layer (from 0–20 cm to 180–200 cm).

ET was determined using the following formula [19]:

ET=P+Δ SWS,

where P was the total precipitation (mm) during the test period. To determine the ET in the cover crop period, P was the precipitation from sowing the rape seed until harvesting the apples. To determine the total ET, P was the total precipitation from bud formation until harvesting the apples. ΔSWS is the change in SWS (mm). For in-row areas, ΔSWS was the change in SWS under the BPM. For inter-row areas, ΔSWS was the change in SWS in the inter-row areas among apple trees.

Nine apple trees were selected randomly from each replicate to assess the apple yield. The yield from each tree was measured by weighing. WUE was calculated as the apple yield divided by ET [19], as follows.

WUE=Yield / ET

The soil temperatures were recorded at depths of 5, 10, 15, 20, and 25 cm at 08:00 h, 10:00 h, 12:00 h, 14:00 h, 16:00 h and 18:00 h using geothermometers (RM-003) during the bud formation stage, cover crop period, and harvest time in 2015. Three sets of instruments were installed for each replicate in the rows of apple trees and in the inter-row areas.

Statistical comparisons were performed between PC and PGC for the in-row and inter-row areas using SPSS 12.0. One-way analysis of variance (ANOVA) was used to detect significance differences in the means of the measured parameters. Means were compared using the Fisher’s Least Significant Difference (LSD) Test at *p* ≤ 0.05.

## Results

### SWS

**Fig 4:**
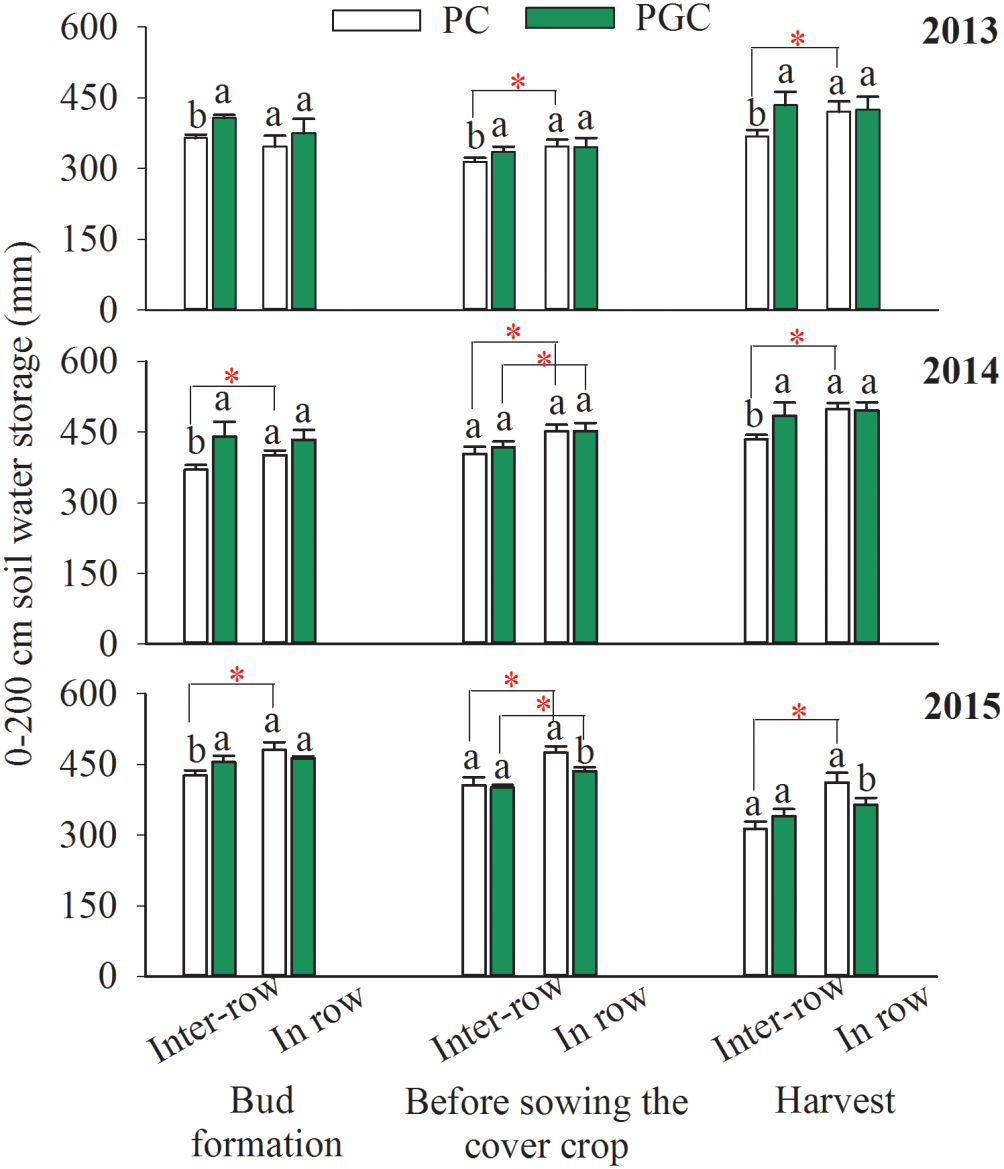
0–200 cm soil water storage in each stage for 2013, 2014 and 2015. Lowercase letters denote significant differences between PC and PGC for the inter-row or in-row (p ≤ 0.05). “*” indicates that the differences between the inter-row and in-row (connected by lines) of the PC or PGC treatment were significant (p ≤ 0.05). Bud formation means the bud formation stage of apple trees (in late March). Before sowing the cover crop refers to the time before sowing the rape (in mid-June). The apple harvest always occurs in early October in this region. Error bars were the standard deviations.

**Fig 5:**
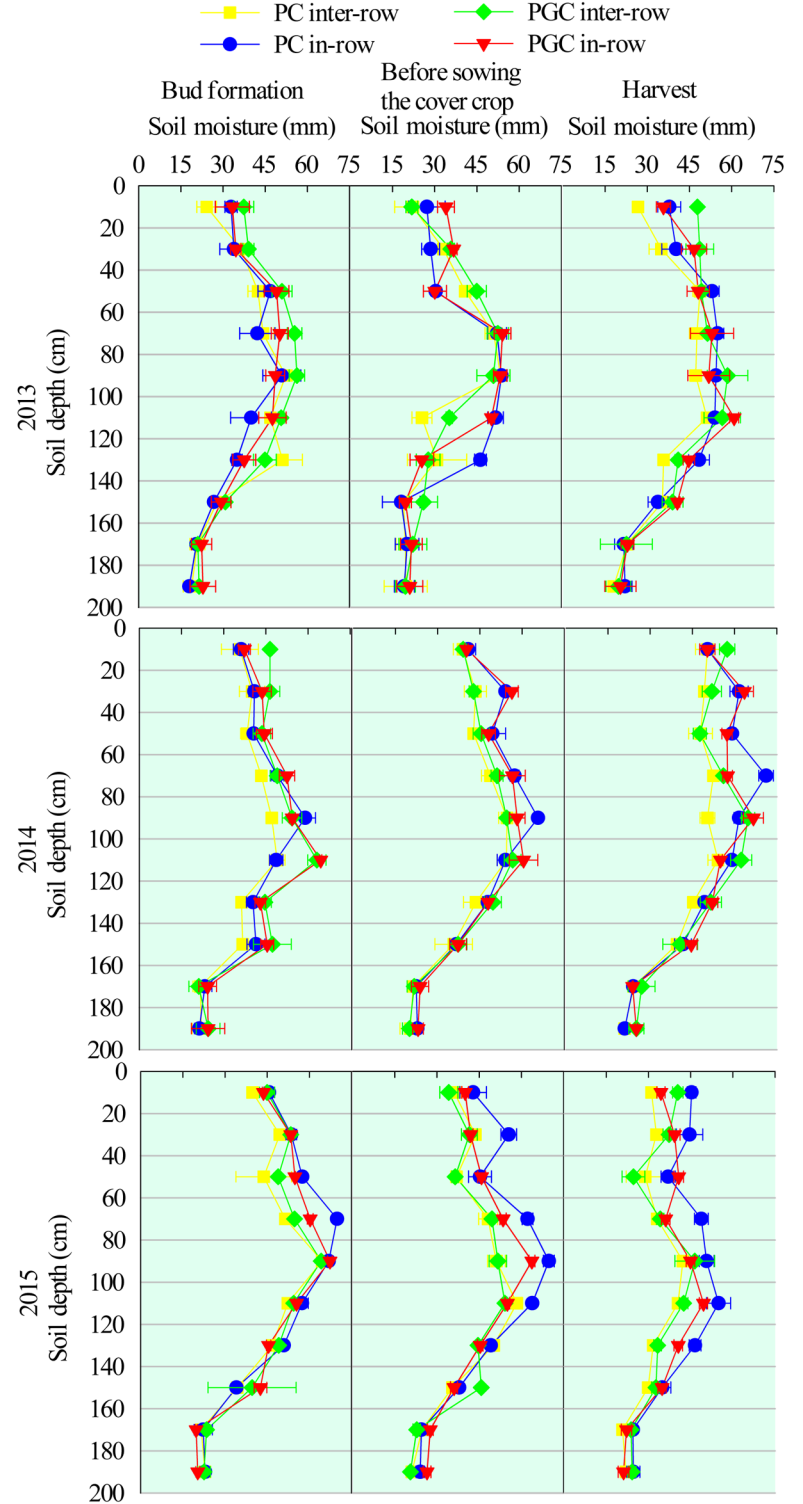
0–200 cm soil moisture (layer by layer) in each treatment (inter-row and in-row) at different stages.

SWS was affected by the distribution of precipitation, air temperature, and water consumption by the apple trees and cover crop. In the bud formation stage, the inter-row SWS was higher under PGC than PC during 2013 (11.8%), 2014 (18.9%), and 2015 (6.7%) (Fig 4), where the differences were caused mainly by increases in the soil water in the soil depth of 0–120 cm each year (Fig 5), whereas the in-row SWS did not differ significantly between the two treatments (Fig 4).

Before sowing the rape, the difference in SWS was not obvious between PC and PGC (significant differences were only found in 2013 for the inter-row SWS and in 2015 for the in-row SWS). However, the inter-row SWS increased compared with the in-row SWS, (except under PGC in 2013) (Fig 4). The increase in the soil water mainly occurred in the soil depth of 0–120 cm each year (Fig 5).

After harvesting, the cover crop had grown for approximately four months in the inter-row areas under the PGC treatment, and the inter-row SWS levels under the PGC treatment were 17.9% and 11.5% higher than those under the PC treatment in 2013 and 2014, respectively (Fig 4), where the differences in the soil water occurred at soil depths of 0–20 and 80–100 cm (Fig 5). Moreover, the in-row SWS was higher under the PC treatment than PGC during 2015 (Figs 4 and 5).

Under the PC treatment, the inter-row SWS was lower than the in-row SWS (the only time that the difference was not significant was during the 2013 bud formation stage) (Fig 4), where the variations in the soil water occurred mainly at the soil depth of 0–140 cm and they decreased from the upper layers to the deeper layers (Fig 5). Under the PGC treatment, significant differences between the inter-row SWS and in-row SWS only occurred before sowing the rape seed in 2014 and 2015 (Fig 4).

### ET during the cover crop period and total ET

**Fig 6:**
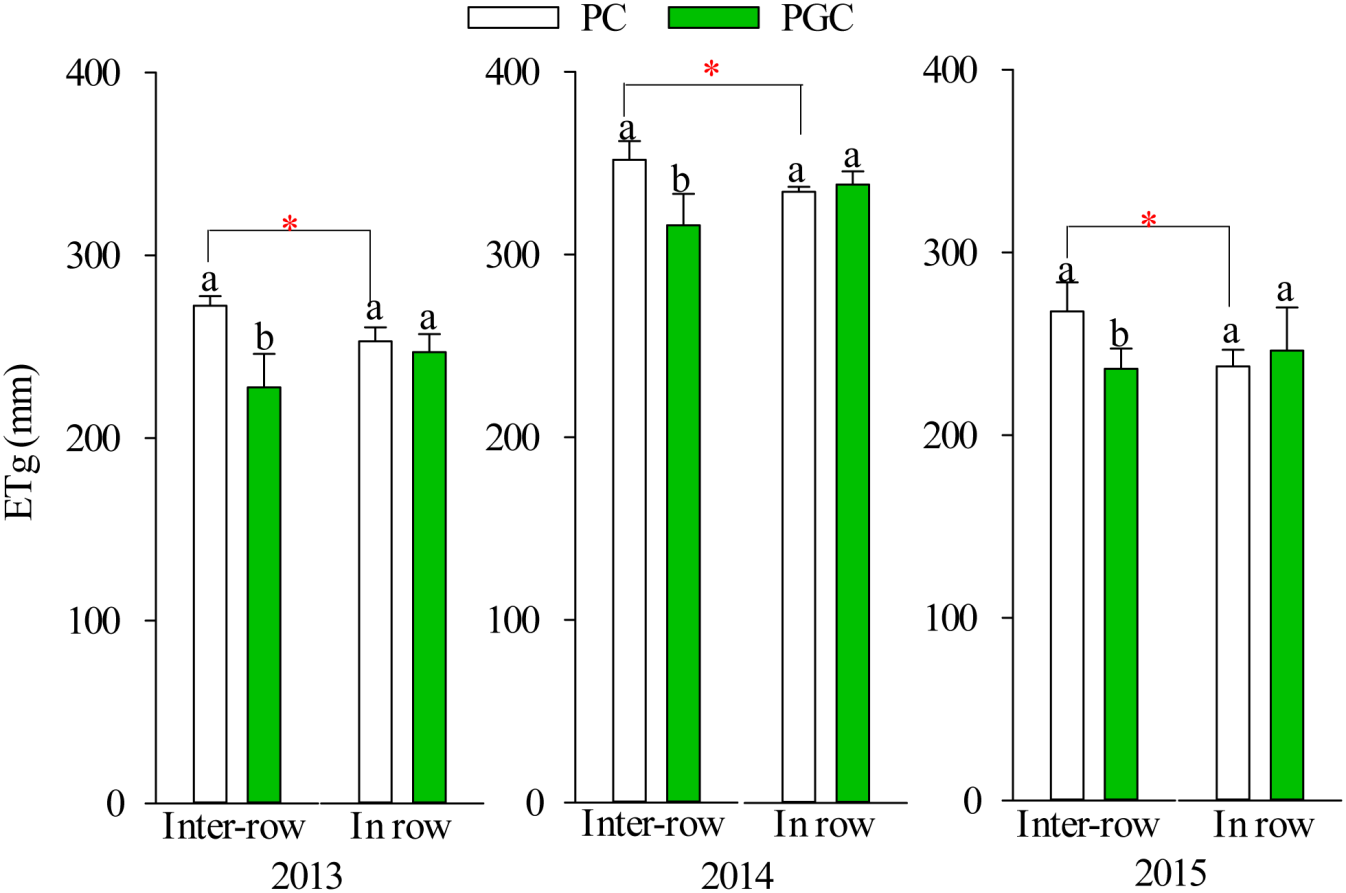
The evapotranspiration during the grass cover period (July-September) in 2013, 2014 and 2015. Lowercase letters denote significant differences between PC and PGC for the inter-row or in-row (*p* ≤ 0.05). “*” indicates that the differences between the inter-row and in-row (connected by lines) of the PC or PGC treatment were significant (*p* ≤ 0.05). Error bars were the standard deviations.

The levels of ET during the cover crop period (ETg) in the inter-row areas were 19.6%, 11.3%, and 13.3% higher under PC than PGC during 2013, 2014, and 2015, respectively, but there were no significant differences in the in-row ETg levels between PC and PGC (Fig 6). Under the PC treatment, the inter-row ETg levels were higher than the in-row ETg during 2013 (7.8%), 2014 (5.1%), and 2015 (12.6%). However, there were no significant differences between the inter-row ETg and in-row ETg under the PGC treatment during the experimental years.

**Fig 7:**
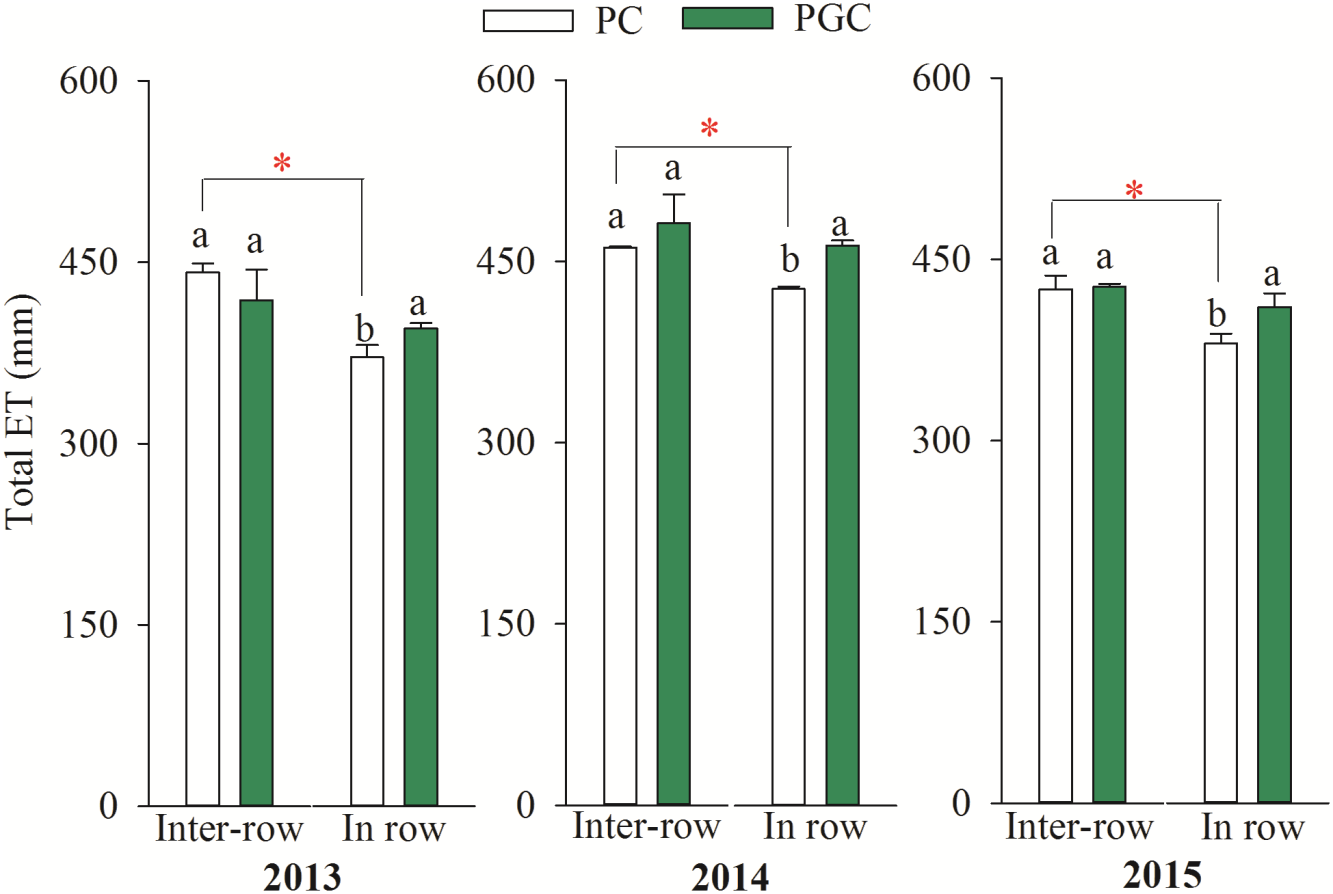
The total evapotranspiration during the growing period (late March to September) of apple trees in 2013, 2014 and 2015. Lowercase letters denote significant differences between PC and PGC for the inter-row or in-row (*p* ≤ 0.05). “*” indicates that the differences between the inter-row and in-row (connected by lines) of the PC or PGC treatment were significant (*p* ≤ 0.05). Error bars were the standard deviations.

There were no significant differences in the total inter-row ET between the two treatments during the three experimental years, where the total in-row ET levels under PGC were 6.4%, 8.4%, and 7.9% higher than those under PC during 2013, 2014, and 2015, respectively (Fig 7). Under the PC treatment, the inter-row ET levels were higher than the in-row ET levels during 2013 (18.9%), 2014 (8.0%), and 2015 (11.8%), respectively, but there were no obvious differences between the inter-row ET and in-row ET levels under the PGC treatment.

**Fig 8:**
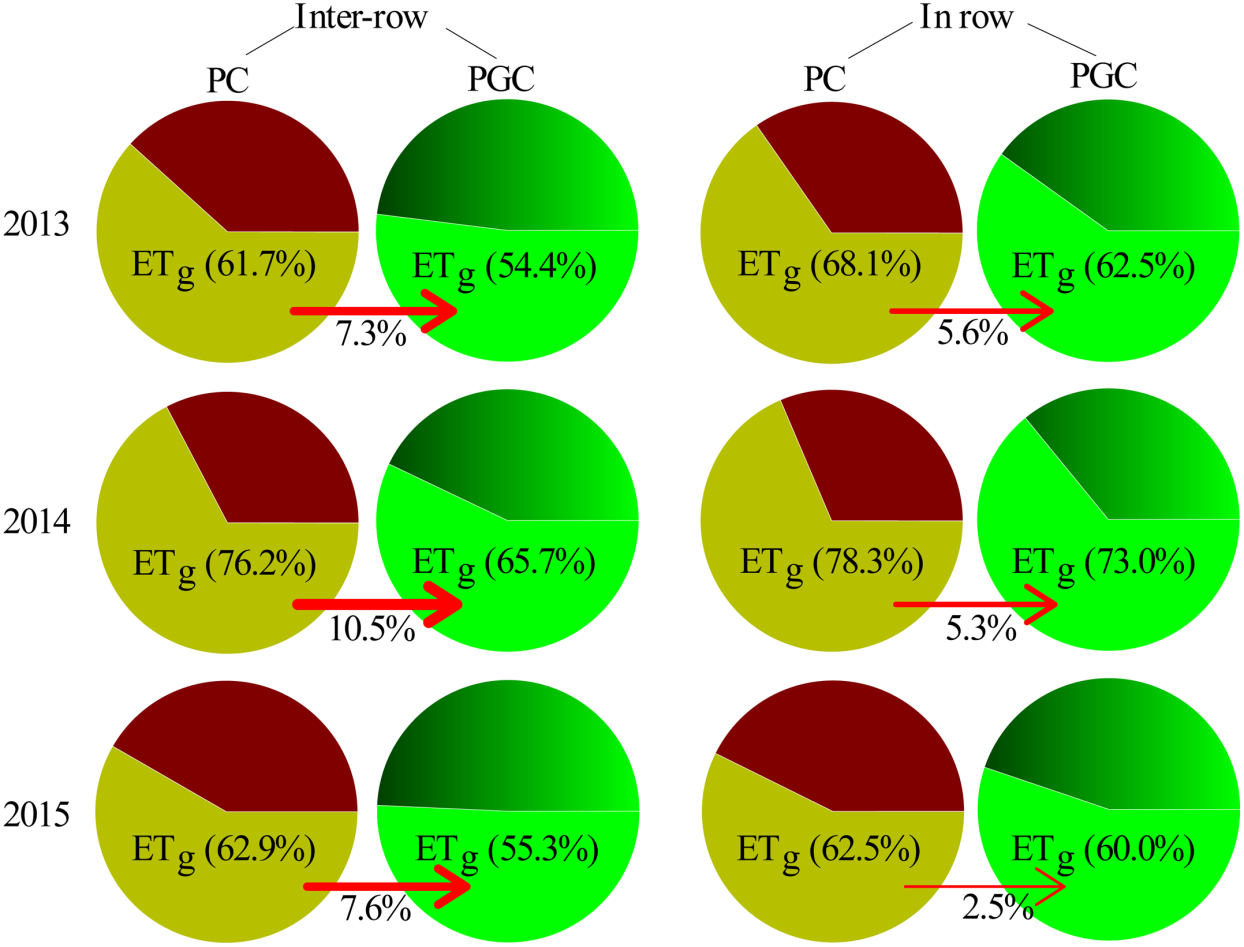
The proportion of evapotranspiration during the grass cover period (ETg) in total evapotranspiration (ETg/ET) in 2013, 2014 and 2015. The red arrow shows the deviations of ETg/ET between PC and PGC for the inter-row and in-row.

The proportion of ETg relative to the total ET (ETg/ET) was higher under PC than PGC in both the inter-row and in-row areas during 2013, 2014, and 2015 (Fig 8). The differences in ETg/ET between PC and PGC in the inter-row areas (7.3% in 2013, 10.5% in 2014, and 7.6% in 2015) were higher than those in the in-row areas (5.6% in 2013, 5.3% in 2014, and 2.5% in 2015).

### Yield, WUE during cover crop period, and total WUE

The highest yield was obtained in 2014 whereas lower yields were obtained in 2013 and 2015 (Table 1). The yields were 14.1%, 18.8%, and 26.7% higher under the PGC treatment than the PC treatment in 2013, 2014, and 2015, respectively (Table 1).

**Table 1:** Yield, water use efficiency during the cover crop period (WUEg), and total water use efficiency (WUE) under different treatments in the apple orchard.

		Yield (kg ha^–1^)	WUEg (kg ha^–1^ mm^–1^)	WUE (kg ha^–1^ mm^–1^)
2013	PC	27135.0 ± 958.9 b	51.6 ± 1.6 b	33.4 ± 0.5 b
	PGC	30960.0 ± 1456.2 a	65.2 ± 0.9 a	38.0 ± 0.9 a
2014	PC	46907.9 ± 1024.1 b	68.3 ± 1.8 b	52.8 ± 1.0 b
	PGC	55746.1 ± 2044.8 a	85.2 ± 2.2 a	59.0 ± 1.1 a
2015	PC	33082.1 ± 4297.4 b	65.4 ± 6.7 b	41.0 ± 4.9 a
	PGC	41909.9 ± 2991.7 a	86.8 ± 0.5 a	50.0 ± 2.9 a
2013	Mean	29047.5 ± 2367.5 C	58.4 ± 7.5 B	35.7 ± 2.6 C
2014	Mean	51327.0 ± 5052.4 A	76.8 ± 9.4 A	55.9 ± 3.5 A
2015	Mean	37496.0 ± 5860.6 B	76.1 ± 12.5 A	45.5 ± 6.1 B

Lowercase letters denote significant differences (*p* ≤ 0.05) between PC and PGC.

Capital letters indicate significant differences (*p* ≤ 0.05) among three years.

Mean values ± standard deviation are shown based on three replicates.

The rates of WUE during the cover crop period (WUEg) were 31.5 and 30.3% higher in 2014 and 2015 than 2013, respectively. The WUEg rates were 26.4%, 24.7%, and 32.7% higher under PGC than PC during 2013, 2014, and 2015, respectively. The highest WUE was obtained in 2014 and lower WUE rates occurred in 2013and 2015 (56.58 and 22.86% lower). The WUE rates were 13.8% and 11.7% higher under PGC than PC during 2013 and 2014, but the difference in 2015 was not significant (*p* = 0.0527).

### Soil temperature

**Fig 9:**
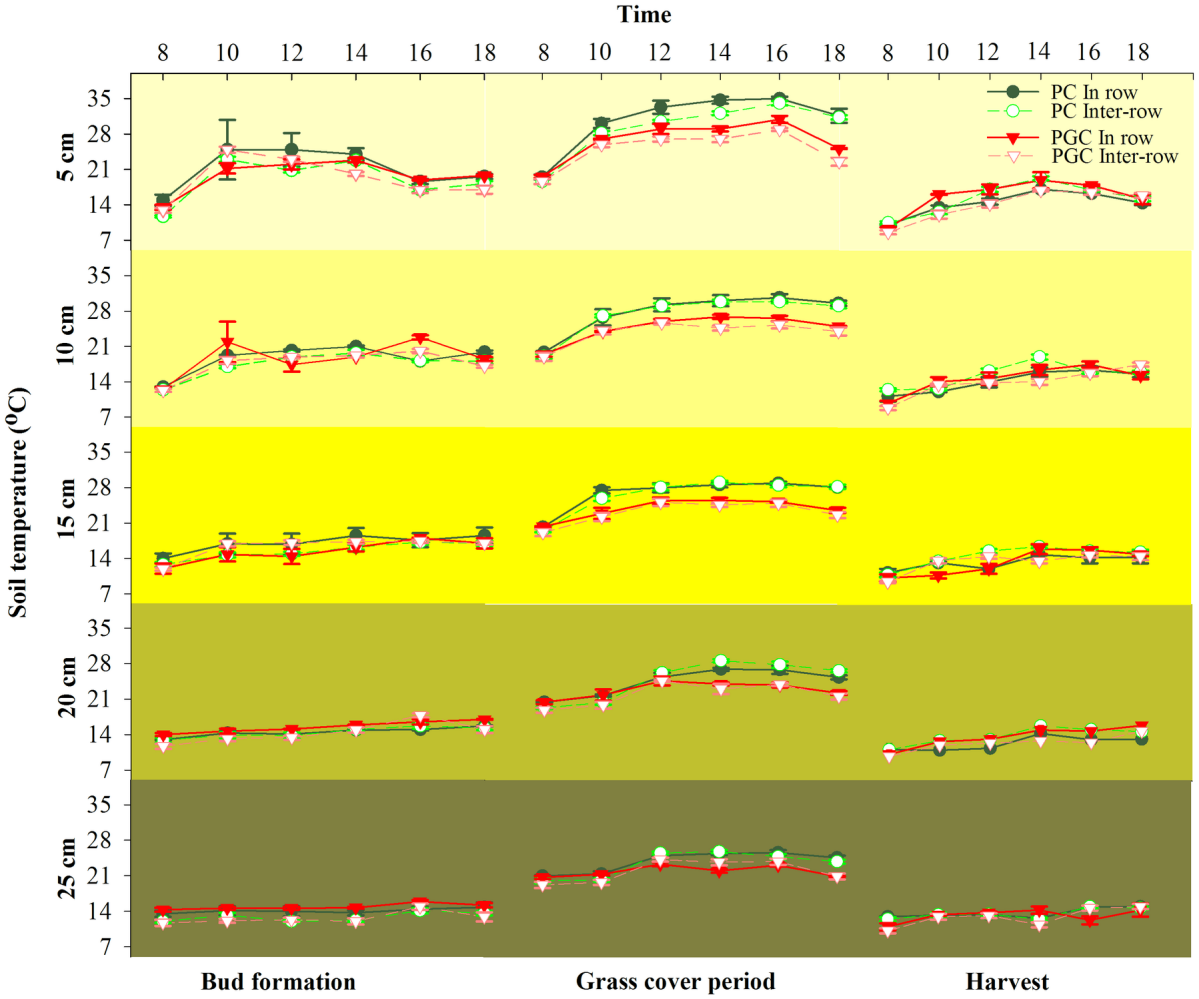
Hourly soil temperature (0–25 cm) during bud formation stage, grass cover period and harvest time in 2015. The soil temperature was recorded from 8:00 to 18:00 every two hours. Bars show standard deviation of the means. Error bars were the standard deviations.

The variations in the soil temperature under different treatments decreased as the soil depth increased (Fig 9). During the bud formation stage and after harvesting, the soil temperature in each layer was lower than 30°C and there were no significant differences between PC and PGC in the inter-row and in-row areas. During the cover crop period, the soil temperature was lower under PGC than PC in both the inter-row and in-row areas, especially at the 5-cm depth at midday and afternoon (Fig 9), where the differences between PC and PGC decreased as the soil depth increased (from 0 to 25 cm).

## Discussion

### Effects of PGC on SWS and ET

PGC increased the inter-row SWS after the harvest compared with PC (Figs 4 and 5), as well as markedly decreasing the ETg, especially in the inter-row areas (Figs 6 and 8). In addition, under the PC treatment, more water was lost in the inter-row areas compared with the in-row areas during the cover crop period, but the difference between the inter-row ETg and in-row ETg was not obvious after applying the cover crop to the inter-row areas under the PGC treatment (Fig 6). These results indicate that the combination of BPM and the cover crop under PGC was more effective than the separate BPM (in-row) with bare soil (inter-row) for conserving water in the hot and rainy summer, which can be explained as follows. The black plastic film (in-row) and cover crop (inter-row) both insulated the soil surface, thereby reducing the exchange of water between the soil and atmosphere, and decreasing evaporation [1,20,21]. The cover crop increased the infiltration of water [11,17] compared with the bare soil [22,23,24]. The cover crop contributed to increases in the soil organic matter content (PC vs. PGC = 12.66 vs. 15.61 g kg^–1^, inter-row in 2015, *p* = 0.023) to improve the soil structure and enhance the water-holding capacity [12,25,26,27,28]. In addition, the cover crop modified the soil temperature [20] and affected soil evaporation [29]. Thus, during a hot summer, the high soil temperature due to strong solar radiation and high shortwave absorbance by the black plastic film [16,30,31] were modified by the presence of the summer cover crop (Fig 9). Other studies have also suggested that bare soil may lose more soil water than covered soil in some situations due to the mulching effect of cover crops [14,15]. Therefore, more water could have been retained in the inter-row areas during the hot summer to support apple growth in the current season as well as allowing more water to be stored to facilitate bud formation in the next season (Figs 4 and 5). However, during the harvest period in 2015, the difference in the inter-row SWS was not significant between PC and PGC (Fig 4), probably because of the lower precipitation during the cover crop period during 2015 (155.2 mm) compared with 2013 (316.2 mm) and 2014 (341.2 mm) (Fig 2).

Furthermore, although the application of BPM combined with a cover crop could conserve more water in the hot summer, the inter-row ET with PGC during the entire growing season was not lower than that with PC, and the in-row ET under PGC was even higher than that under PC (Fig 7). In addition, under the PC treatment, the total inter-row ET was higher than the total in-row ET, which did not occur under the PGC treatment. It is likely that more water could be retained under the PGC treatment, where the cover crop (after being incorporated into the soil) employed in the PGC treatment could supply more nutrients to promote tree growth, improve the apple yield, and increase the water consumption by apple trees.

In this experiment, we considered whether the cover crop might compete for soil water with fruit trees, as found in other studies [32,33,34,35,36], but this did not occur (Figs 4 and 5), probably because the cover crop was only applied in the rainy season, and competition between the cover crop and apple trees could have been mitigated by the abundant precipitation. In addition, compared with the bare soil, the water consumed by the cover crop was compensated for by its mulching effect in the hot and rainy season (Fig 6).

### Response of yield and WUE to the combination of BPM and summer cover crop

During the experimental years, the apple yield, WUEg and WUE fluctuated due to biennial bearing. The application of BPM combined with a cover crop was more efficient than BPM alone in terms of increasing the apple yield, WUEg, and WUE, although the increase in WUE was not obvious during 2015 (Table 1). However, previous studies in a vineyard [17,18,37], apricot orchard [36], red birch nursery [38], and apple orchard [39] found that the application of a cover crop could affect tree growth and the fruit yield via water competition, which contrasted with our results. This difference may be explained by the lack of competition for water between the cover crop and apple trees in our experiment, as explained above. In addition, the apple trees were likely to seek other soil zones located principally below the tree rows and in deeper layers due to the competition between trees and the cover crop, as shown previously by Smith et al. [40] with a *Grevillea robusta—Zea mays* agroforestry system, Celette et al. [41] with a *Vitis vinifera* L.–*Festuca arundinacea* Shreb. intercropping system, Fetene [42] with an *Acacia etbaica—Hyparrhenia hirta* association, Lehmann et al. [43] with an *Acacia saligna—Sorghum bicolor* association, and Parker and Meyer [44] with *Prunus persica* L.–*Paspalum notatum* Flugge, and *Bromus mollis* L. and *Eremochloa ophiuroides* (Munro) Hack. associations. Thus, the competition for soil resources between two species could be limited by the adaptive spatial complementarity of their root systems [41]. In addition, the cover crop could have acted as a green manure after being incorporated into the soil, thereby improving the soil fertility and supplying more nutrients for tree growth [45,46,47,48,49] compared with the application of BPM alone. Therefore, the apple yield, WUEg, and WUE were increased under PGC compared with PC in our experiment, although the difference in the WUE between PC and PGC was not significant during 2015 (*p* = 0.0527).

## Conclusions

As expected, compared with the use of BPM alone, the combined application of BPM and a cover crop increased the inter-row SWS after the harvest and decreased the summer ETg in the inter-row areas, although the total in-row ET was increased due to more water being consumed by the apple trees under PGC during the whole growing season. Thus, the apple yield under PGC was not affected by water consumption by the cover crop and it was higher than that under PC. Moreover, the WUEg increased under PGC during the experimental years compared with PC. The total WUE was also increased by PGC, although the effect was not obvious in 2015. In addition, the application of the cover crop under PGC during a hot summer modified the high temperature due to strong solar radiation and BPM. Hence, the application of BPM combined with a cover crop is expected to be a beneficial practice for farmers engaged in apple production on the Loess Plateau and other similarly arid and semiarid areas.
